# Effects of (S)-ketamine on depression-like behaviors in a chronic variable stress model: a role of brain lipidome

**DOI:** 10.3389/fncel.2023.1114914

**Published:** 2023-02-15

**Authors:** Cuihong Zhou, Xinxin Zhao, Xinxu Ma, Hongzhe Ma, Rui Li, Guangtao Hu, Huaning Wang, Zhengwu Peng, Min Cai

**Affiliations:** ^1^Department of Psychiatry, Xijing Hospital, The Fourth Military Medical University, Xi’an, Shaanxi, China; ^2^Department of Anesthesiology and Perioperative Medicine, Xijing Hospital, The Fourth Military Medical University, Xi’an, Shaanxi, China; ^3^Department of Psychological Medicine, 958th Hospital, Chongqing, China

**Keywords:** CVS: chronic variable stress, (S)-ketamine, major depressive disorder, lipidomic, hippocampus, prefrontal cortex

## Abstract

**Introduction:** Compelling evidence indicates that a single sub-anesthetic dose of (S)-ketamine elicits rapid and robust antidepressant effects. However, the underlying mechanisms behind the antidepressant effects of (S)-ketamine remain unclear.

**Methods:** Here, using a chronic variable stress (CVS) model in mice, we analyzed changes inthe lipid compositions of the hippocampus and prefrontal cortex (PFC) with a mass spectrometry-based lipidomic approach.

**Results:** Similar to previous research outcomes, the current study also showed that (S)-ketamine reversed depressive-like behaviors in mice produced by CVS procedures. Moreover, CVS induced changes inthe lipid compositions of the hippocampus and PFC, notably in the contents of sphingolipids, glycerolipids, and fatty acyls. With the administration of (S)-ketamine, CVS-induced lipid disturbances were partially normalized, particularly in the hippocampus.

**Conclusion:** Altogether, our results indicated that (S)-ketamine could rescue CVS-induced depressive-like behaviors in mice through region-specific modulation of the brain lipidome, contributing to the understanding of (S)-ketamine’s antidepressant effects.

## 1. Introduction

Major depressive disorder (MDD) is a life-threatening mental disorder identified by low mood, anhedonia, inappropriate sense of guilt, cognitive disturbances, andsuicidal thoughts and behaviors (Smith, 2014). More than 350 million people suffer from MDD, with a prevalence rate of about 5.7% worldwide (Ferrari et al., 2013). For many years, studies mainly focused on monoaminergic antidepressants in the treatment of MDD due to the relatively well-accepted monoamine hypothesis of depression (Bristow et al., 2021; Jiang et al., 2022). However, monoaminergic antidepressants show limitations in the clinic, such as a delayed onset of response, inevitable side effects, and partial efficacy (Machado-Vieira et al., 2017). Therefore, it is even more challengingto explore novel therapeutic candidates with different mechanisms of action.

Unlike monoaminergic antidepressants, a single treatment of NMDAR receptor antagonist (R,S)-ketamine at a sub-anesthetic dose has been found to elicit rapid and sustained antidepressant effects in MDD patients and also depressive-like animal models (Berman et al., 2000; Zanos et al., 2016; Yang et al., 2018; Simmler et al., 2022). As the S enantiomer of (R,S)-ketamine, (S)-ketamine has been approved by the US Food and Drug Administration (FDA) for antidepressant use and showed a rapid antidepressant effect among patients with treatment-resistant depression and reduced suicide ideation in MDD patients (Cristea and Naudet, 2019). Moreover, recent evidence from an open-label study showed that intranasal (S)-ketamine had long-term safety for patients with treatment-resistant depression (Canuso et al., 2018; Daly et al., 2019). The most recent investigation also reported that (S)-ketamine reduced *I*_h_ or prevented *I*_h_ from increasing following chronic unpredictable stress and upregulated the sirtuin type 1 (SIRT1)-mediated BDNF expression, which could be responsible for (S)-ketamine’s rapid antidepressant effects (Kim and Johnston, 2020; Hou et al., 2022). However, mechanisms underlying the antidepressant effects of (S)-ketamine remain underexplored.

In the central nervous system (CNS), lipid populations play an increasingly recognized role in the maintenance and modulation of energy metabolism, synaptic plasticity, neurotransmission, and regulation of multiple signaling pathways (Hussain et al., 2019; Glombik et al., 2022). These cascades of biological processes influenced by lipids are associated with a range of emotional behaviors and cognitive functions (Hashimoto et al., 2015; Egawa et al., 2016). Abnormal lipidomic alterations in the brain have been reported in relevance with numerous CNS diseases and mental disorders, such asanxiety disorder, schizophrenia, and MDD (Muller et al., 2015; Kavoor et al., 2017; Yamamura et al., 2021). Specifically, it has been indicated that alterations in brain lipids and lipid composition participatein the pathogenesis of MDD (Ong et al., 2016; Knowles et al., 2017; Peng et al., 2018). Changes in lipid metabolism were also detected in relation to the clinical response of antidepressant treatment in patients with treatment resistant depression (TRD; Bekhbat et al., 2018). In line with these clinical investigations, preclinical researches using rodent depressive models demonstrated that membrane lipid composition could be altered by long-term antidepressant use (McIntyre et al., 2010; Kivity et al., 2017). Overall, existing evidence strongly indicates that changes in lipid composition could be the exact underlying mechanism of the rapid antidepressant effect of (S)-ketamine, but further investigation is still needed.

In this study, we applied a chronic variable stress model to induce depressive-like behaviors in order to clarify the changes in lipid composition in the hippocampus and PFC, two of the most important brain regions in the pathogenesis and treatment of MDD in mice treated with a single *i.p.* injection of (S)-ketamineafter 3 weeks of CVS exposure.

## 2. Materials and methods

### 2.1. Animals

Animals used in this study were 8 weeks old male C57BL/6 mice (weighing 18–22 g). Mice were group-housed five per cage in wire-bottomed cages at 20–25°C with 12 h light/12 h dark daily cycle (lights on from 8:00 AM to 8:00 PM), and allowed to consume food and water freely after being obtained from the Fourth Military Medical University Animal Center (Xi’an, China). Experiments were conducted during the light phase in accordance with the National Institutes of Health Guide for the Care and Use of Laboratory Animals and approved by the Animal Use and Protection Committee of the Fourth Military Medical University.

### 2.2. Experimental design

Seven days after acclimatization, mice were randomly assigned to the following three groups: Control (*n* = 10), CVS + saline (*n* = 10), and CVS + (S)-Ket (*n* = 11). Mice in the CVS + saline and CVS + (S)-Ket groups were subjected to the CVS protocol for 21 consecutive days and then received *i.p.* injections of either saline or 10 mg/kg (S)-ketamine once respectively. Twenty-four hours after the *i.p.* injections, the depressive-like behaviors were measured through an open field test, novelty suppressed feeding test, forced swimming test, and tail suspension test. After those behavioral tests, the mice were sacrificed, and the PFC and hippocampus tissues were isolated from the mice brain and stored in liquid nitrogen immediately for later lipidomic analysis using high-performance liquid chromatography-mass spectrometry (HPLC-MS).

### 2.3. The establishment of depressive-like behavior by chronic variable stress procedure

The depressive-like behavior was produced by a chronic variable stress protocol reported by a previous study (Bittar et al., 2021). Briefly, mice were subjected to three different stressors repeated for 21 days. On day 1, mice were stimulated with 100 foot-shocks at 0.45 mA randomly for 1 h. On day 2, mice were suspended by their tail for a 1-h operation. On day 3, mice were restrained in a 50 ml falcon tube for 1 h. Over 21 days, mice were repeatedly subjected to these stressors once per 3 days.

### 2.4. Drug administration

(S)-ketamine (25 mg/ml, Hengrui Medicine Co., Ltd) was diluted in saline to a working solution of 1.0 mg/ml. Mice were weighed and treated with (S)-ketamine at a 10 mg/kg dose or saline vehicle controls at a dosage of 200 μl per 20 g mouse by intraperitoneal (*i.p.)* injections.

### 2.5. Behavioral testing

Behavioral experiments occurred 24 h after the i.p injection in the following order: novelty suppressed feeding test (NSFT), open field test (OFT), tail suspension test (TST), and forced swim test (FST). In order to minimize stress-related anxiety effects, mice were acclimated to the experimental room for at least 60 min prior to tests, and all experiments were conducted under dim lighting conditions, and the testing area was cleaned with 75% ethanol between trials.

#### 2.5.1. Open-field test (OFT)

In a previous study, mice were placed and approved to freely move in the open-field box (50 × 50 × 50 cm; Muir et al., 2020). The track of each mouse was recorded for a period of 5 min by a video camera positioned directly above the arena and analyzed by the open-field activity software Top Scan (Clever Sys Inc., USA). The floor of the open field box was divided into 36 squares including 16 central and 20 peripheral squares. Finally, the distance traveled and time spent both in the central squares and the entire arena was measured.

#### 2.5.2. Novelty suppressed feeding test (NSFT)

NSFT was confirmed in accordance to a published protocol (Bittar et al., 2021). Mice were food-restricted 24 h prior to the test. Then, mice were habituated to the testing room and placed into an open field box (50 × 50 × 50 cm) which was covered with the bedding at the bottom and a single pellet of food was placed in the center.

The video camera positioned directly above the arena was used to record the whole process of the experiment. Mice were allowed to explore the box and eat under red-light conditions until they started to eat or when the 10-min test time ended.

#### 2.5.3. Tail suspension test (TST)

During the tail suspension test, mice were hung to a horizontal bar using a standard laboratory tape adhered to their tails. Mice were hung for 6 min elevated 50 cm above the floor, and activity was recorded by a video camera. Immobility, defined as lack of skeletal movement for at least 1 s, was analyzed for the duration of the last 5 min of the 6 min test *via* automated tracking software (Freeze Scan, Clever Sys, Inc.). Any mouse that crawled back up its tail was removed from the data analysis (Eagle et al., 2020).

#### 2.5.4. Forced swim test (FST)

A forced swimming test was confirmed based on a previous study with minor modifications (Lei et al., 2020). In brief, each mouse was placed into a Plexiglas cylinder (inner diameter, 14 cm; 30 cm high) containing water (20 cm of water; 23°C–25°C) and was allowed to swim freely for 6 min. The activity of each mouse was recorded by a video camera, and the immobility time during the last 5 min of the 6-min test was measured by automated tracking software (Freeze Scan, Clever Sys, Inc.). The cylinder was cleaned and then refilled with fresh water before the next test.

### 2.6. Tissue collection

All the mice were euthanized by cervical dislocation after the behavioral test. The PFC and hippocampus were isolated and dissected on ice immediately, then precisely weighed, frozen, and stored in liquid nitrogen until lipidomic analysis was performed by high-performance liquid chromatography-mass spectrometry (HPLC-MS).

### 2.7. Sample extraction

Lipidome quantification and data analysis were established as the description in a previous study and technically supported by Shanghai Applied Protein Technology Co., Ltd. Lipids were extracted with methyl tert-butyl ether (MTBE) as previously described (Cajka et al., 2017; Jiang et al., 2017). Briefly, 30 mg samples were spiked with appropriate amounts of internal lipid standards which include 13 isotope mixtures of internal lipid standards, SPLASH^®^ LIPIDOMIX MASS SPRC STANDARD, AVANTI, 330707-1EA, and homogenized with 240 μl of methanol and 200 μl of water. Subsequently, samples were spiked with 800 μl of MTBE and ultrasonicated at 4°C for 20 min, then incubated at 25°C for 30 min. The organic components were separated from the solution by centrifuging at 10°C for 15 min at 14,000× *g* to the. The upper organic solvent phase was collected and evaporated under nitrogen. Finally, the solutes were stored at −80°C before use.

### 2.8. Lipid analysis by HPLC–MS/MS

The ultra-HPLC separation was performed through reverse-phase chromatography by using a CSH C18 column (ACQUITY UPLC CSH C18, 1.7 μm, 2.1 × 100 mm, Waters). The column temperature was 45°C, and the flow rate was 300 μl/min. The extraction of lipid was centrifuged at 14,000 × *g* for 15 min after re-dissolved in 200 μl of 90% isopropanol/acetonitrile; then, 3 μl of each sample was perfused for analysis. The initial mobile phase was 30% solvent B (consists of acetonitrile–isopropanol (1:9, v/v), 0.1% formic acid, and 0.1 mM ammonium format) at a flow rate of 300 μl/min for 2 min, then linearly increased to 100% solvent B over 23 min, followed by equilibration in 5% solvent B for 10 min. The autosampler was maintained at 10°C. Multiple samples were analyzed concurrently, and signal fluctuations were detected in random order, alsoduring sample analysis, to avoid bias due to instrumentation errors. A sampling of the queue was confirmed every eighth sample using one of the quality control (QC) samples to observe the stability of the analysis and measure the reliability of the experimental data.

Next, samples were analyzed by a Q Exactive^TM^ plus mass spectrometer (Thermo Fisher Scientific, Waltham, USA) after being separated by ultra-HPLC. The parameters were set as following: positive-ion mode:heater temperature, 300°C; sheath gas flow rate, 45 arbitrary units (arb); auxiliary gas flow rate, 15 arb; sweep gas flow rate, 1 arb; spray voltage, 3.0 kV; capillary temperature, 350°C; S-Lens radio frequency (RF) level, 50%; and MS1 scan range, 200–1,800 m/z; negative-ion mode: heater temperature, 300°C; sheath gas flow rate, 45 arb; auxiliary gas flow rate, 15 arb; sweep gas flow rate, 1 arb; spray voltage, 2.5 kV; capillary temperature, 350°C; S-Lens RF level, 60%; and MS2 scan range, 250–1,800 m/z.

### 2.9. Lipid identification through lipid search^TM^

Lipid species were identified with a search engine LipidSearch system (Thermo Fisher Scientific, Walthamcity, country, USA) based on MS/MS database. Over 30 lipid classes and more than 1,500,000 ion fragments were included in this database. The mass tolerances for molecular precursors and fragment ions were set to 5 ppm. The displayed product ion threshold was set at five, and all four grades (A-D) applied in the identification (ID) quality filtering. All 71 sub-species of the lipid classes in the database were selected for identification. Adducts containing H^+^ and NH_4_^+^ were chosen for positive-mode searches, and adducts containing H^−^ and CH_3_COO^−^ werechosen for negative-mode searches since ammonium acetate was applied for the mobile phases.

### 2.10. Statistical analysis

The statistical analyses in the current study were confirmed by SPSS v.21.0 software (SPSS Inc., Chicago, IL, USA). All data of behavioral testing and the characterizations of lipid compositions were expressed as mean ± standard deviation (SD), and were evaluated by one-way analysis of variance (ANOVA) with Bonferroni *post-hoc* test for pairwise comparisons. The two-tailed *P*-value < 0.05 was considered as statistically significant. The correlations between lipid species levels and behaviors were analyzed using the Pearson correlation.

The peak recognition, lipid-peak extraction (secondary appraisal), peak alignment, and quantitative processing of raw lipidomic data were processed using LipidSearch software. After normalizing to the total peak intensity and integrating with the Pareto scaling method, the processed data were imported into SIMPCA-P 14.1 (Umetrics, Umea, Sweden) for multivariate statistical analysis, including principal component analysis, partial least squares discriminant analysis, and orthogonal partial least squares discriminant analysis. The quality of OPLS-DA was checked using a permutation test. In the same time, the variable importance in the projection (VIP) values was obtained from each lipid variable to examine the contribution of variables, and the Student’s *t*-test followed by the adjustment of the false discovery rate (FDR) in multiple hypothesis tests was introduced to obtain the *P* values of each variable. Lipids with significant differences were identified based on a combination of fold change (> 1.5 or < 0.67), Student’s *t*-test FDR < 0.01, and VIP > 1 in according to the methods from our previous reports (Xue et al., 2020; Zhou et al., 2022).

## 3. Results

### 3.1. (S)-ketamine ameliorates depression-like behaviors in mice exposed to CVS

**Figure 1:**
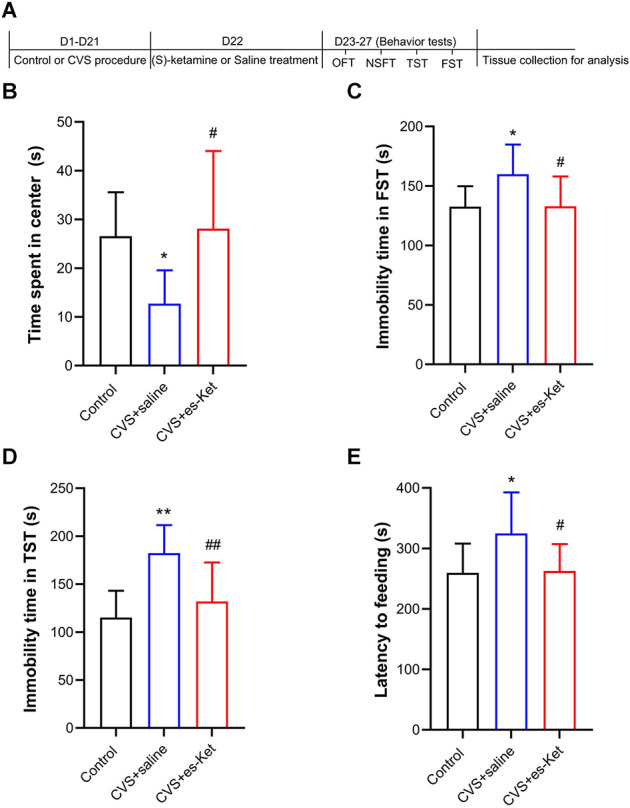
(S)-Ketamine ameliorates depressive-like behaviors in chronic variable stress (CVS) treated mice. **(A)** Schedule for stress, treatment, and behavioral tests. **(B)** Time spent in the center squares of OFT (*F*_2, 28_ = 5.555, *P* = 0.009). **(C)** Immobility time in the FST (*F*_2, 28_ = 4.751, *P* = 0.017). **(D)** Immobility time in the TST (*F*_2, 28_ = 10.93, *P* < 0.001). **(E)** Latency time to feeding in the NSFT (*F*_2, 28_ = 4.628, *P* = 0.018). *P < 0.5 vs. Control; **P < 0.01 vs. Control; ^#^*P* < 0.05 vs. CVS + saline; ^##^*P* < 0.01 vs. CVS + saline.

As illustrated in Figure 1, we first identified the effect of (S)-ketamine on depression-like behaviors. The One-way ANOVA results indicated that there were significant differences in the time spent in the center squares of the OFT (*F_2, 28_* = 5.555, *P* < 0.01), latency to feeding in NSFT (*F_2, 28_* = 4.629, *P* < 0.05), as well as immobility time in both TST (*F_2, 28_* = 10.930, *P* < 0.01) and FST (*F_2, 28_* = 4.751, *P* < 0.05) between the three groups. *Post hoc* comparisons further revealed that CVS markedly reduced the time spent in the central arena in the OFT, and these behavioral outcomes were ameliorated in (S)-ketamine-treated group [CVS + (S)-Ket *vs*. CVS + saline, *P* < 0.05]. Additionally, the CVS + saline group showed a significant increase in latency to feeding as well as immobility time in both TST and FST when compared with the Control group (*P* < 0.01). Whereas, the increased latency to food, the increased immobility time in FST and TST produced by the CVS procedure were reversed by (S)-ketamine administration [CVS + (S)-Ket *vs*. CVS + saline, *P* < 0.05].

### 3.2. Identification of the number of lipid compounds

Based on the criterion from the International Lipid Classification and Nomenclature Committee, lipid compounds are classified into eight types. Moreover, each class type can be split into different subtypes with polarity as the head of the class (lipid class). Based on the different saturabilities or lengths of the carbon chain, each subgroup was classified into different molecular species (lipid species) to achieve a three-level classification of lipid compounds. As shown in Supplementary Figure 1, we finally identified 1,006 lipid species and 42 lipid classes in the samples of each group.

### 3.3. The impact of (S)-ketamine on the hippocampal lipids

**Figure 2:**
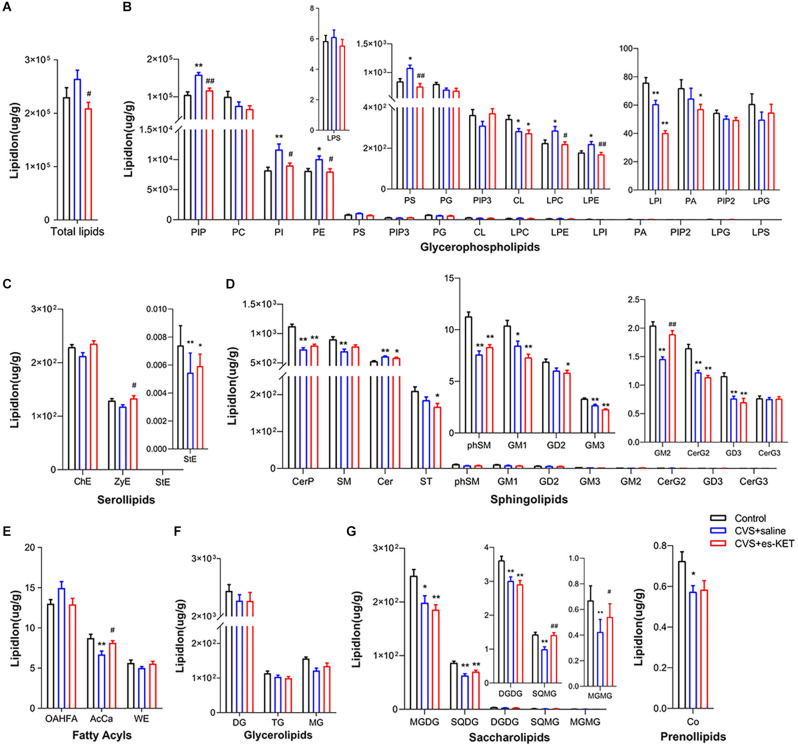
Effect of (S)-Ketamine on the lipidomic profiles in the hippocampus of CVS-treated mice. **(A)** Total lipid concentration, **(B)** glycerophospholipids, **(C)** serollipids, **(D)** sphingolipids, **(E)** fatty acyls, **(F)** glycerolipids, **(G)** saccharolipids and prenol lipids. PIP, phosphatidylinositol phosphate; PC, phosphatidylcholine; PI, phosphatidylinositol; PE, phosphatidylethanolamine; PS, phosphatidylserine; PIP3, phosphatidylinositol 3,4,5-trisphosphate; PG, phosphatidylglycerol; CL, cardiolipin; LPC, lysophosphatidylcholine; LPE, lysophosphatidylethanolamine; LPI, lysophosphatidylinositol; PA, phosphatidic acid; PIP2, phosphatidylinositol (4,5) bisphosphate; LPG, lysophosphatidylglycerol; LPS, lysophosphatidylserine; ChE, Cholesterol Ester; ZyE, zymosterol; StE, Stigmasterol ester; CerP, Ceramides phosphate; SM, sphingomyelin; Cer, ceramides; ST, Sulfatide (galactosyl ceramide sulfate); phSM, phytosphingosine sphingomyelin; GM1, gangliosides; GD2, Ganglioside (disialodihexosyl ceramide); GM3, Ganglioside (monosialotrihexosyl ceramide); GM2, Ganglioside (monosialodihexosyl ceramide); CerG2, CerG2GNAc1 (N-acetylhexosyl ceramide); GD3, Ganglioside (disialotrihexosyl ceramide); CerG3, CerG3GNAc1 (Dihexosyl N-acetylhexosyl ceramide); OAHFA, (O-acyl)-1-hydroxy fatty acid; AcCa, acylcarnitine; WE, wax esters; DG, diglyceride; TG, triglyceride; MG, monoglyceride; MGDG, monogalactosyldiacylglycerol; SQDG, sulfoquinovosyldiacylglycerol; DGDG, digalactosyldiacylglycerol; SQMG, Sulfoquinovosylmonoacylglycerol; MGMG, monogalactosylmonoacylglycerol; Co, coenzyme; CVS, chronic variable stress; es-Ket, (S)-ketamine; *P < 0.05 *vs.* Control; **P < 0.01 *vs.* Control; ^#^*P* < 0.05 *vs.* CVS + saline; ^##^*P* < 0.01 *vs.* CVS + saline.

As shown in Figure 2 and Supplementary Table 1, we identified significant intergroup differences in total lipid levels and the concentrations of 29 lipid classes, such as phosphatidylinositol phosphate (PIP), phosphatidylethanolamine (PE), and gangliosides (GM2). *Post hoc* analysis revealed that mice in the CVS group exhibited significantly elevated levels of PIP, phosphatidylinositol (PI), phosphatidylethanolamine (PE), phosphatidylserine (PS), lysophosphatidylcholine (LPC), lysophosphatidylethanolamine (LPE) and Ceramides (Cer), but reduced levels of cardiolipin (CL), lysophosphatidylinositol (LPI), stigmasterol ester (StE), ceramides phosphate (CerP), sphingomyelin (SM) sphingomyelin phytosphingosine (phSM), gangliosides (GM1, GM3, GM2, GD3), simple glc series (CerG2NAc1), acyl carnitines (AcCa), monogalactosyldiacylglycerol (MGDG), sulfoquinovosyldiacylglycerol (SQDG), digalactosyldiacylglycerol (DGDG), sulfoquinovosylmonoacylglycerol (SQMG), monogalactosylmonoacylglycerol (MGMG), and coenzyme (Co) (CVS + saline *vs.* Control, *P* < 0.05, respectively). Esketamine treatment significantly reduced the levels of PIP, PI, PE, PS, LPC, and LPE, and up-regulated the levels of GM2, AcCa, SQMG, and MGMG [CVS + (S)-Ket vs. CVS + saline, *P* < 0.05]. However, the changes in CL, LPI, CerP, SM, Cer, phSM, GM1, GM3, CerG2GNAc1, GD3, MGDG, SQDG, DGDG, and Co produced by CVS in the hippocampus were not normalized by the administration of (S)-ketamine.

**Figure 3:**
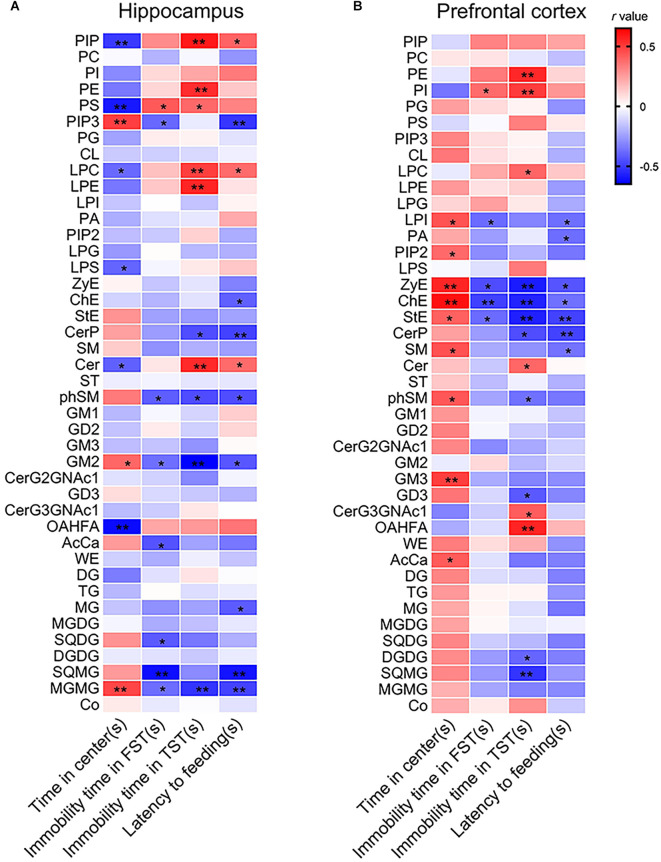
Pearson’s correlation analysis between depressive-like behaviors and lipid levels in **(A)** the hippocampus and **(B)** the PFC. *P < 0.05; **P < 0.01.

Correlation analysis indicated that the time spent in the center arena in the OFT was positively in relation tolevels of phosphatidylinositol 3,4,5-trisphosphate (PIP3), GM2, and MGMG but negatively correlated with levels of PIP, PS, LPC, LPS, Cer, and (O-acyl)-1-hydroxy fatty acid (OAHFA). The immobility time in the TST was positively correlated with levels of PIP, PE, PS, LPC, LPE, and Cer but negatively correlated with levels of CerP, phSM, GM2, and MGMG. Moreover, the latency time to feeding in the NSFT was positively correlated with levels of PIP, LPC, and Cer but negatively correlated with levels of PIP3, cholesterol Ester (ChE), CerP, phSM, GM2, MG, SQMG, and MGMG (Figure 3A and Supplementary Table 2).

### 3.4. The impact of (S)-ketamine on the prefrontal cortex lipids

**Figure 4:**
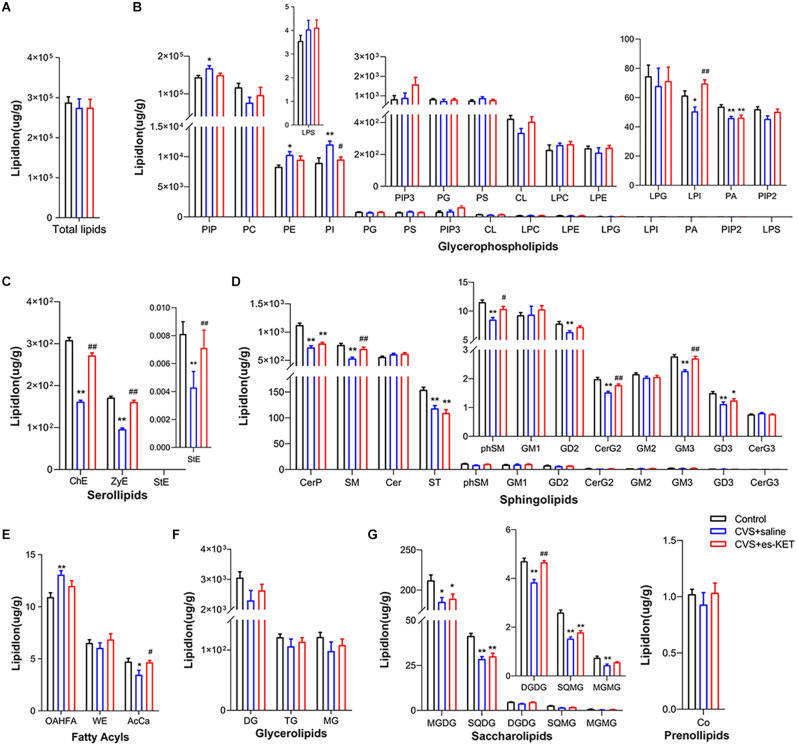
Effect of (S)-Ketamine on the lipidomic profiles in the prefrontal cortex (PFC). **(A)** Total lipid concentration, **(B)** glycerophospholipids, **(C)** serollipids, **(D)** sphingolipids, **(E)** fatty acyls, **(F)** glycerolipids, **(G)** saccharolipids and prenol lipids. PIP, phosphatidylinositol phosphate; PC, phosphatidylcholine; PI, phosphatidylinositol; PE, phosphatidylethanolamine; PS, phosphatidylserine; PIP3, phosphatidylinositol 3,4,5-trisphosphate; PG, phosphatidylglycerol; CL, cardiolipin; LPC, lysophosphatidylcholine; LPE, lysophosphatidylethanolamine; LPI, lysophosphatidylinositol; PA, phosphatidic acid; PIP2, phosphatidylinositol (4,5) bisphosphate; LPG, lysophosphatidylglycerol; LPS, lysophosphatidylserine; ChE, Cholesterol Ester; ZyE, zymosterol; StE, Stigmasterol ester; CerP, Ceramides phosphate; SM, sphingomyelin; Cer, ceramides; ST, Sulfatide (galactosyl ceramide sulfate); phSM, phytosphingosine sphingomyelin; GM1, gangliosides; GD2, Ganglioside (disialodihexosyl ceramide); GM3, Ganglioside (monosialotrihexosyl ceramide); GM2, Ganglioside (monosialodihexosyl ceramide); CerG2, CerG2GNAc1 (N-acetylhexosyl ceramide); GD3, Ganglioside (disialotrihexosyl ceramide); CerG3, CerG3GNAc1 (Dihexosyl N-acetylhexosyl ceramide); OAHFA, (O-acyl)-1-hydroxy fatty acid; AcCa, acylcarnitine; WE, wax esters; DG, diglyceride; TG, triglyceride; MG, monoglyceride; MGDG, monogalactosyldiacylglycerol; SQDG, sulfoquinovosyldiacylglycerol; DGDG, digalactosyldiacylglycerol; SQMG, Sulfoquinovosylmonoacylglycerol; MGMG, monogalactosylmonoacylglycerol; Co, coenzyme; CVS, chronic variable stress; es-Ket, (S)-ketamine; *P < 0.05 *vs.* Control; **P < 0.01 *vs.* Control; ^#^*P* < 0.05 *vs.* CVS + saline; ^##^*P* < 0.01 *vs.* CVS + saline.

As shown in Figure 4 and Supplementary Table 1, no statistical intergroup differences were found in total lipid levels in the prefrontal cortex (Figure 4A). However, significant differences were observed in concentrations of 23 lipids between the three groups, such as LPI, SM, and phSM in this structure. The results of *post-hoc* comparisons showed that mice in the CVS group exhibited significantly increased levels of PIP, PE, PI, and OAHFA, but reduced levels of LPI, PA, ChE, zymosterol (ZyE), stigmasterol ester (StE), CerP, SM, ST, phSM, GD2, CerG2GNAc1, GM3, GD3, AcCa, MGDG, SQDG, DGDG, SQMG, and MGMG CVS + saline *vs.* Control, *P* < 0.05). (S)-ketamine administration effectively decreased the levels of PI and increased the levels of LPI, ChE, ZyE, StE, SM, phSM, CerG2GNAc1, GM3, AcCa, and DGDG [CVS + (S)-Ket *vs*. CVS + saline, *P* < 0.05]. However, the changes in PIP, PE, PA, CerP, ST, GD2, GD3, OAHFA, MGDG, SQDG, SQMG, and MGMG produced by CVS expose in the prefrontal cortex were not normalized by the treatment of (S)-ketamine.

The results of correlation analysis showed that the time spent in the center arena in the OFT was positively in correlation with levels of LPI, phosphatidylinositol (4,5) bisphosphate (PIP2), ZyE, ChE, StE, SM, phSM, GM3, and AcCa. The immobility time of the TST was positively in correlation with levels of PI but negatively correlated with levels of LPI, ZyE, ChE, and StE. The immobility time in the TST was positively correlated with levels of PE, PI, LPC, Cer, CerG3GNAc1, and OAHFA but negatively correlated with levels of ZyE, ChE, StE, CerP, GD3, DGDG, and SQMG. Moreover, the latency time to feeding in the NSFT was negatively correlated with levels of LPI, PA, ZyE, ChE, StE, CerP, and SM (Figure 3B and Supplementary Table 3).

### 3.5. The impact of (S)-ketamine on the fatty acid composition

**Figure 5:**
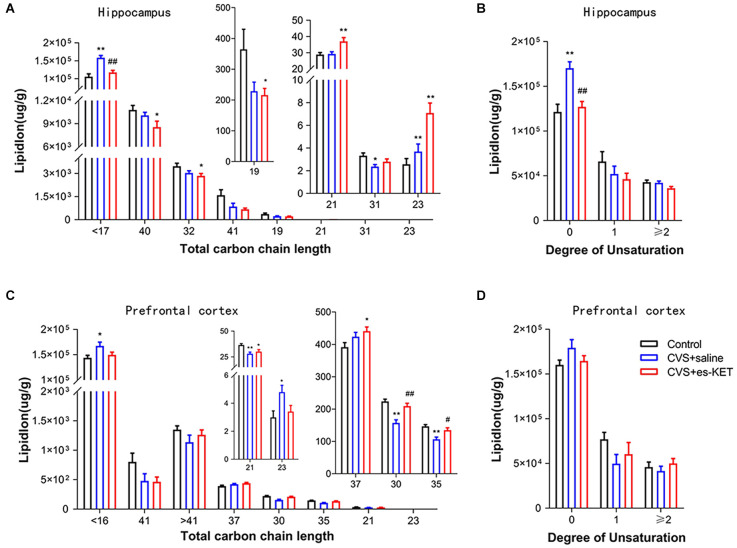
Effect of (S)-Ketamine on the fatty acid composition in the hippocampus and PFC in CVS-treated mice. Fatty acyl composition was analyzed by **(A,C)** chain length (number of carbons) totals and **(B,D)** degree of unsaturation totals. CVS, chronic variable stress; es-Ket, (S)-ketamine; *P < 0.05 *vs.* Control; **P < 0.01 *vs.* Control; ^#^*P* < 0.05 *vs.* CVS + saline; ^##^*P* < 0.01 *vs.* CVS + saline.

As illustrated in Figure 5 and Supplementary Table 9, CVS exposure significantly altered lipids’ fatty acyl chain profile in the hippocampus and PFC. In the hippocampus, levels of long-chain fatty acyls with less than 17 carbons (<17C) and 23 carbons (23C), and levels of saturated fatty acyls (Figure 5B) were increased. In contrast, levels of long-chain fatty acyls with 31 carbons (31C) were decreased in CVS + saline group (CVS + saline *vs.* Control, *P* < 0.05). (S)-ketamine administration normalized the levels of long-chain fatty acyls with less than 17 carbons and saturated fatty acyls [CVS + (S)-Ket*vs*. CVS + saline, *P* < 0.05]. Meanwhile, in the PFC, levels of long-chain fatty acyls with < 16 carbons (16C) and 23 carbons (23C) were increased, while levels of long-chain fatty acyls with 21 carbons (21C), 30 carbons (30C), and 35 carbons (35C) were decreased in CVS + saline group (CVS + saline *vs.* Control, *P* < 0.05). (S)-ketamine administration normalized the levels of long-chain fatty acyls with 30 and 35 carbons [CVS + (S)-Ket vs. CVS + saline, *P* < 0.05]. We did not find significate differences in chain saturation in the PFC between the three groups (Figure 5D).

### 3.6. The characteristic of lipid species in the brain and their correlation with depressive-like behaviors

Additional intergroup changes in the concentrations of lipids both in the hippocampus and PFC were found at the species level according to lipidomic profiling. Data of lipids in the hippocampus and PFC could be well distinguished between the three groups by the characteristics of the PLS-DA model (Supplementary Figures 2A,D). Differential species were determined based to the following criteria: (1) FC > 1.5 or < 0.67; and (2) *P* value < 0.05. Altogether, 95 lipid species were dysregulated after CVS, and 19 of them were rescued by (S)-ketamine administration in the hippocampus (Supplementary Figures 2B,C). A total of 61 lipid species were dysregulated after CVS, and five of them were normalized after the administration of (S)-ketamine in the PFC (Supplementary Figures 2E,F).

**Figure 6:**
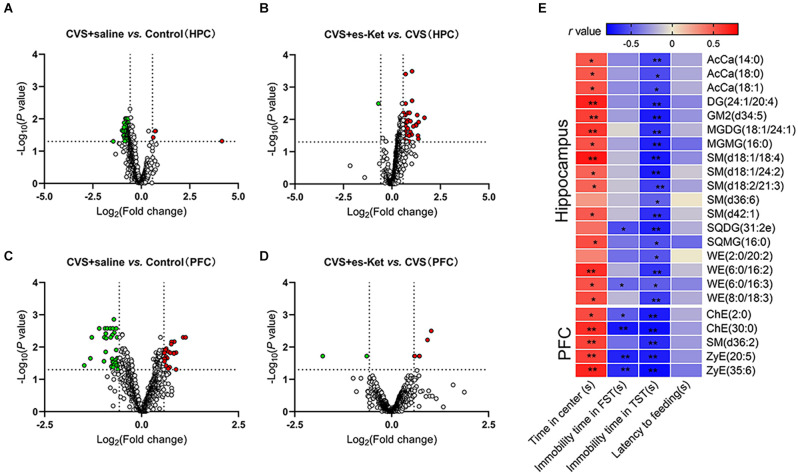
Alterations of lipid species and their correlations with depressive-like behaviors. **(A,B)** Volcano map showing altered (green dots, decreased; red dots, increased) lipid species comparing the CVS + saline group and Control, the CVS + es-Ket group, and the CVS + saline group in the hippocampus. **(C,D)** Same comparisons were for the PFC. The vertical dotted lines in the graph means Log_2_ (0.667) and Log_2_ (1.5), the horizontal dotted line means -Log_10_ (0.05). **(E)** Results of Pearson’s correlation between depressive-like behaviors and lipid species levels in the hippocampus and PFC. PFC, prefrontal cortex; *P < 0.05; **P < 0.01.

As illustrated in Figure 6 and Supplementary Table 4, the level of 91 lipids such as PC (36:4), MGDG (34:5), and AcCa (14:0) were decreased. Four lipids, including Cer (d17:1/18:0), LPC (14:0), and PE (16:0/20:4), were elevated in the hippocampus in the CVS + Saline group compared with the Control group (Figure 6A). (S)-ketamine treatment increased 44 lipids such as AcCa (14:0), PC (16:0/18:3), and SM (d42:2) and decreased Cer (d17:1/18:0) in the hippocampus in CVS-treated mice (Figure 6B). Especially, 19 of the 95 lipid species changed in the hippocampus of CVS-exposed mice, for example, Cer (d17:1/18:0), AcCa (14:0), and SM (d42:1) normalized after (S)-ketamine supplementation (see Supplementary Table 5 for details).

Furthermore, the result of correlation analysis (Figure 6E) showed that the concentrations of 14 lipids including AcCa (14:0), DG (24:1/20:0), and SM (d18:1/18:4) in the hippocampus were positively correlated with the time spent in the center squares in the OFT, but negatively correlated with immobility time in the TST. Moreover, concentrations of SQDG (31:2e) and WE (6:0/16:3) in the hippocampus were also negatively correlated with the immobility time in the FST (see Supplementary Table 6 for details).

Similar to the results in the hippocampus, the PFC analysis revealed changes in the lipid concentrations in multiple species following the CVS procedure (Figures 6C,D, and Supplementary Table 7). Generally, the levels of 38 lipids including PC (6:0/12:4), ChE (2:0), and SM (d36:2) were decreased, meanwhile, the levels of 23 other lipids, such as Cer (m44:3), LPC (24:0), and PE (11:0/10:1) were increased in the PFC of CVS + saline mice compared with Control (Figure 6C). (S)-ketamine treatment increased the levels of six lipids such as ZyE (35:6), ChE (2:0), and SM (d36:2), and decreased the levels of PI (18:0/20:3) and PE (18:2e/20:1) compared with the CVS + saline group (Figure 6D). Notably, levels of 5 of the 61 lipid molecules in the PFC changed after CVS, including ChE (30:0), SM (d36:2), and ZyE (20:5). However, these dysfunctions were rescued after (S)-ketamine treatment (as shown in Supplementary Table 5 for details).

Correlation analysis further revealed that concentrations of ChE (2:0), ChE (30:0), SM (d36:2), ZyE (20:5), and ZyE (35:6) in the PFC were positively correlated with time spent in the central zone in the OFT, but negatively correlated with immobility time in the TST. In addition, four of those lipids were also negatively correlated with immobility time in the FST (Figure 6E and Supplementary Table 8).

## 4. Discussion

In the present study, we performed comprehensive lipid profiling based on HPLC-MS/MS to analyze the effect of (S)-ketamine treatment on lipid modulation in the hippocampus and PFC of mice subjected to CVS. Depressive-like behaviors and lipid changes were detected in the brain of mice exposed to CVS. Notably, a single dose of (S)-ketamine administered intraperitoneally rapidly reversed CVS-induced depressive-like behaviors and partially normalized CVS-induced lipid changes, which were more prominent in the hippocampus. These findings revealed that dysfunction of the brain lipidome in certain brain regions might participate in the pathogenesis of MDD, and modulation of brain lipidome in the hippocampus and PFC might partly contribute to the rapid anti-depressive effect of (S)-ketamine.

A single sub-anesthetic dose of (R,S)-ketamine (0.5 mg/kg with *i.v.* injection for consecutively 40 min) has been found to produce rapid (hours) and sustained (days to weeks) antidepressant effects in TRD and bipolar disorder patients (Murrough et al., 2013, 2015; Grunebaum et al., 2018). Moreover, a series of clinical trials have identifiedthe efficacy and safety of intranasal (S)-ketamine (Daly et al., 2018, 2019; Popova et al., 2019). Therefore, the FDA approved this treatment for TRD and MDD with suicidal thoughts. In animal studies, the supplementation of a single sub-anesthetic dose of (R,S)-ketamine (10–20 mg/kg i.p.) elicits rapid and sustained antidepressant-like effects. Consistent with the antidepressant-like effect produced by (R,S)-ketamine, Chung Sub Kim et al. and other groups reported that a single dose of (S)-ketamine was capable of producing rapid and sustained antidepressant effect (Bristow et al., 2021). In line with the previous reports, we also demonstrated that a single administration of (S)-ketamine produced an antidepressant-like effect in OFT, NSFT, FST, and TST. Despite the compelling evidence supporting (S)-ketamine’s rapid and robust antidepressant effect in clinical and preclinical studies, the mechanisms underlying its antidepressant actions are still unclear.

In CNS, the hippocampus and PFC are two crucial regions involved in emotional regulation (Sanacora et al., 2012). Atrophy of the hippocampus is one of the most significant pathologic changes in MDD patients and rodent depressive-like models (Chen et al., 2010; Kabra et al., 2022). However, improvement in MDD symptoms as a result of antidepressant medications or other maneuvers, such as ketamine administration, have been observed to restore hippocampal structure and function (Duan et al., 2013; Ardalan et al., 2017). PFC emerged as another crucial region consistently impaired in MDD. The functional, structural, and system-level abnormalities of PFC have been reported in both preclinical and clinical studies (Wang et al., 2021; Pizzagalli and Roberts, 2022). Various treatment interventions, including ketamine, have been observed to mechanically rescue PFC dysregulation (Zhou et al., 2020; Ma et al., 2022). In addition, regulation of the PFC-hippocampus circuit has been shown to be associated with the rapid onset of ketamine’s antidepressant effect (Carreno et al., 2016). Therefore, in the present study, we hypothesized that altered lipid composition in the hippocampus and PFC might be linked to the depressive-like behaviors produced by CVS and the antidepressant effect of (S)-ketamine. We found that after CVS procedure, concentrations of 29 lipid classes, such as PIP and PI, changed in the hippocampus, while concentrations of 23 lipid classes, such as PI and OAHFA, changed in the PFC. Moreover, current research found that supplementation of (S)-ketamine partly rescued 10 out of 29 lipid changes in the hippocampus produced by CVS procedure and 11 out of 23 lipid changes in PFC. This suggests that changes in lipid composition induced by stress or (S)-ketamine is region-specific, which may be relevant to the different structures and functions between brain regions.

The lipids, predominantly composed of glycerophospholipids, sphingolipids, and cholesterol, play a crucial role in regulating cell signaling cascades (Muller et al., 2015; Muallem et al., 2017). In membranes of mammalian cells, phosphatidylcholine (PC), PE, PS, PA, and PI are considered typical glycerophospholipids. Meanwhile, cardiolipin (CL) and phosphatidylglycerol (PG) are classified as major polyglycerophospholipids. Changes in the levels of these lipids, such as PC and Cer, were detected in brain tissues from the autopsy specimens of patients with bipolar disorders or schizophrenia (Schwarz et al., 2008). Furthermore, an abundance of LPC, PE, and PI in the plasma of MDD patients was found to be positively related to the severity of depression symptoms (Liu et al., 2016). SM constitutes the majority of cellular sphingolipid and can be hydrolyzed into Cer by acid sphingomyelinase (ASM). Recent studies indicated that increased activity of the ASM/Cer system could be interconnected with the dysregulation of inflammation response and oxidative stress, and this system showed its potential as a novel antidepressant target (Kornhuber et al., 2014; Kurz et al., 2019). Additionally, GM1 is a crucial factor in regulating mammalian neuronal functions, and CL plays a key role in maintaining energy metabolism and mitochondrial function (Chiricozzi et al., 2020; Meng et al., 2020). Moreover, the homeostasis of LPC is vital to myelination in the brain (Plemel et al., 2018). PE is involved in the regulation of membrane fluidity and protection of cells against oxidative stress. Previous studies from our group and others reported that the anti-depressants and non-pharmacological neuromodulation maneuvers regulated the specific glycerophospholipids and sphingolipids levels in both hippocampus and PFC. In accordance with these findings, our research observed that exposure to the CVS procedure significantly increased the levels of PIP, PI, PE, PS, LPC, LPE, and Cer, but reduced the expression of CL, LPI, StE, CerP, SM, phSM, gangliosides, CerG2NAc1, MGDG, SQDG, DGDG, SQMG, MGMG, and Co in the hippocampus. (S)-ketamine treatment rescued the changed levels of PIP, PI, PE, PS, LPC, LPE, GM2, SQMG, and MGMG in the hippocampus. Additionally, in the PFC, a significant increase in PIP, PE, and PI levels were detected, while LPI, PA, ChE, zymosterol (ZyE), stigmasterol ester (StE), CerP, SM, ST, phSM, GD2, CerG2GNAc1, GM3, GD3, MGDG, SQDG, DGDG, SQMG, and MGMG levels decreased. The reduction in PI expression and increase in LPI, ChE, ZyE, StE, SM, phSM, CerG2GNAc1, GM3, and DGDG levels were normalized in PFC after (S)-ketamine administration. We also conducted a correlation analysis to further explore the relevance between depressive-like behaviors and changes in lipids. The results showed that hippocampal levels of phSM, GM2, and MGMG were negatively correlated, while levels of PIP, PS, LPC, and Cer were positively correlated with depressive-like impairments. In PFC, the levels of LPI, PIP2, ZyE, ChE, StE, SM, phSM, and GM3 were negatively associated, while the levels of PE, PI, LPC, Cer, CerG3GNAc1, and OAHFA were positively correlated with depressive-like behaviors.

Furthermore, a growing body of evidence showed that dysfunction in glycolipid regulation played a vital role in the pathophysiology of MDD. In the brain, AcAa compounds are crucial for stabilizing the plasma membrane, which maintains mitochondrial function and antioxidant capacity (Tarasenko et al., 2018). Some studies have demonstrated that the plasma level of AcCa is correlated with the severity of depressive symptoms in subjects with MDD (Cassol et al., 2015). Our results showed a significant decrease in the abundance of AcCa in both hippocampus and PFC of mice, which was normalized by (S)-ketamine treatment. This indicates that the rescued AcAc level correlates with the antidepressant effect of (S)-ketamine.

Chain length (number of carbons) and the degree of saturation of the constituent fatty acyls can also influence the biophysical properties of lipids. In CNS, neuronal functions could be affected by the modulation of fatty acyl chains and the degree of saturation of membrane lipids. The current study found that changes in fatty acyls composition in the hippocampus attributable to CVS could be ameliorated after (S)-ketamine treatment. These changes in PFC were more extensive and could not be normalized by (S)-ketamine, suggesting that fatty acyl composition in the PFC is more susceptible to CVS-produced stress and cannot be rescued to the same extent as hippocampus fatty acyl composition.

Lastly, some limitations of this study should be noted. First, one single dose of (S)-ketamine injection has been demonstrated to elicit rapid and sustained antidepressant effect, however, the normalized lipidome was either more in relation to the rapid antidepressant effect or its sustained response of (S)-ketamine still need more clarification. Although the current study has confirmed the correlation analysis between changes in lipid content and behavioral outcomes and found that numerous lipid molecule levels were related to depressive-like behaviors, the underlying mechanisms of (S)-ketamine-introduced antidepressant behavior, such as BDNF-TrkB signaling cascade and the normalized lipid abundance remain unclear.

In summary, the current study identified distinct changes in lipid composition in the hippocampus and PFC in mice after CVS exposure. Furthermore, it was found that lipids in the hippocampus were more sensitive to CVS influence than those in the PFC. Additionally, the administration of (S)-ketamine could partially normalize the changes in lipid composition, specifically in sphingolipids and glycerophospholipids. Taken together, these outcomes indicate that altered lipid levels across the hippocampus and PFC may be responsible for the depressive-like behaviors induced by CVS and the antidepressant effect of (S)-ketamine.

## Data availability statement

The original contributions presented in the study are included in the article/Supplementary material, further inquiries can be directed to the corresponding author/s.

## Ethics statement

The animal study was reviewed and approved and all animal-related procedures were approved by the Ethics Committee for Animal Experimentation of the Fourth Military Medical University (Xi’an, China) and proceeded in accordance with the National Institute of Health Guide for the Care and Use of Laboratory Animals (NIH Publications No. 80-23) revised 1996 or the ARRIVE Guidelines for the Care and Use of Laboratory Animals.

## Author contributions

CZ: experimental design, methodology, investigation, writing, and funding acquisition. XZ: experimental design, methodology, data collection, and investigation. XM: data collection and investigation. HM: data collection, investigation, and writing. RL: data collection and investigation. GH: data collection, investigation, and supervision. HW: experimental design, supervision, and funding acquisition. ZP: experimental design, investigation, writing, project administration, and funding acquisition. MC: experimental design, investigation, writing, project administration, and funding acquisition. All authors contributed to the article and approved the submitted version.

## Figures and Tables

**Table:** 

PFC	prefrontal cortex
CVS	chronic variable stress
HPLC–MS/MS	high performance liquid chromatography–tandem mass spectrometry
OFT	open field test
EPMT	elevated-plus maze test
FST	forced swim test
TST	tail suspension test
MTBE	methyl tert-butyl ether
SD	standard deviation
ANOVA	one-way analysis of variance
DG	diglyceride
TG	triglyceride
SM	sphingomyelin
Cer	ceramides
GM1	gangliosides
GD2	ganglioside
CerG3GNAc1	Dihexosyl N-acetylhexosyl ceramide
CerG2GNAc1	N-acetylhexosyl ceramide
OAHFA	(O-acyl)-1-hydroxy fatty acid
CL	cardiolipin
LPG	lysophosphatidylglycerol
LPI	lysophosphatidylinositol
LPS	lysophosphatidylserine
PA	phosphatidic acid
PE	phosphatidylethanolamine
PG	phosphatidylglycerol
PI	phosphatidylinositol
AcCa	acylcarnitine
SIRT1	sirtuin type 1
SQDG	sulfoquinovosyldiacylglycerol
SQMG	sulfoquinovosylmonoacylglycerol
TRD	treatment resistance depression
MGMG	monogalactosylmonoacylglycerol
MGDG	monogalactosyldiacylglycerol
Co	coenzyme
So	sphingosine
DGDG	digalactosyldiacylglycerol
LPC	lysophosphatidylcholine
PC	phosphatidylcholine
WE	wax esters
phSM	sphingomyelin
PS	phosphatidylserine
CerG1	glucosylceramides
ChE	cholesterol ester
StE	stigmasterol ester
ZyE	zymosterol
