# Supplementary material for: Effects of (S)-ketamine on depression-like behaviors in a chronic variable stress model: a role of brain lipidome
Source: Front Cell Neurosci. 2023 Feb 15;17:1114914. doi: 10.3389/fncel.2023.1114914 (PMC9975603; doi:10.3389/fncel.2023.1114914)
Supplement: Supplementary file 1 [file Table_1.DOCX]

**Table S1. Effect of CVS on the concentration of lipids in the hippocampus and prefrontal cortex**

| lipids | Hippocampus | | Prefrontal cortex | |  |
| --- | --- | --- | --- | --- | --- |
|  | *F _(2,28)_* | *P* | *F _(2,28)_* | *P* |  |
| Total lipids | 3.389 | 0.048 | 0.142 | 0.869 |  |
| AcCa | 6.776 | 0.004 | 4.999 | 0.014 |  |
| WE | 1.306 | 0.287 | 0.777 | 0.469 |  |
| OAHFA | 2.537 | 0.097 | 5.589 | 0.008 |  |
| DG | 0.631 | 0.540 | 0.150 | 0.862 |  |
| TG | 0.161 | 0.852 | 0.219 | 0.805 |  |
| MG | 0.130 | 0.878 | 0.141 | 0.869 |  |
| StE | 6.795 | 0.004 | 31.540 | <0.001 |  |
| ZyE | 3.865 | 0.033 | 167.000 | <0.001 |  |
| ChE | 3.084 | 0.062 | 111.500 | <0.001 |  |
| CL | 5.298 | 0.011 | 2.762 | 0.080 |  |
| LPC | 5.068 | 0.013 | 0.773 | 0.471 |  |
| LPE | 6.191 | 0.006 | 0.647 | 0.531 |  |
| LPG | 2.908 | 0.071 | 1.825 | 0.180 |  |
| LPI | 39.260 | <0.001 | 10.690 | <0.001 |  |
| LPS | 0.440 | 0.648 | 0.849 | 0.438 |  |
| PA | 4.745 | 0.017 | 8.591 | 0.001 |  |
| PC | 2.103 | 0.141 | 1.492 | 0.242 |  |
| PG | 3.103 | 0.061 | 0.666 | 0.522 |  |
| PI | 7.073 | 0.003 | 6.205 | 0.006 |  |
| PS | 9.874 | <0.001 | 1.507 | 0.239 |  |
| PE | 5.473 | 0.009 | 3.492 | 0.044 |  |
| PG | 3.103 | 0.061 | 0.666 | 0.522 |  |
| PIP | 16.040 | <0.001 | 4.063 | 0.028 |  |
| PIP2 | 1.888 | 0.170 | 2.906 | 0.071 |  |
| PIP3 | 1.790 | 0.186 | 2.284 | 0.121 |  |
| Cer | 10.220 | <0.001 | 2.074 | 0.145 |  |
| CerG2 | 30.840 | <0.001 | 17.790 | <0.001 |  |
| CerG3 | 0.050 | 0.951 | 1.389 | 0.266 |  |
| CerP | 46.920 | <0.001 | 46.920 | <0.001 |  |
| GD2 | 4.533 | 0.020 | 5.498 | 0.009 |  |
| GD3 | 16.840 | <0.001 | 7.502 | 0.003 |  |
| GM1 | 12.830 | <0.001 | 1.868 | 0.173 |  |
| GM2 | 24.350 | <0.001 | 1.319 | 0.284 |  |
| GM3 | 34.180 | <0.001 | 17.170 | <0.001 |  |
| SM | 7.220 | 0.003 | 15.670 | <0.001 |  |
| phSM | 29.720 | <0.001 | 11.330 | <0.001 |  |
| ST | 4.667 | 0.018 | 16.110 | <0.001 |  |
| DGDG | 9.328 | <0.001 | 18.360 | <0.001 |  |
| MGDG | 8.044 | 0.002 | 5.174 | 0.012 |  |
| MGMG | | 13.470 | <0.001 | 7.515 | 0.002 |
| SQDG | | 13.300 | <0.001 | 17.890 | <0.001 |
| SQMG | | 10.950 | <0.001 | 42.160 | <0.001 |
| Co | | 3.994 | 0.030 | 0.446 | 0.644 |
