# Supplementary material for: Effects of (S)-ketamine on depression-like behaviors in a chronic variable stress model: a role of brain lipidome
Source: Front Cell Neurosci. 2023 Feb 15;17:1114914. doi: 10.3389/fncel.2023.1114914 (PMC9975603; doi:10.3389/fncel.2023.1114914)
Supplement: Supplementary file 2 [file Table_2.DOCX]

**Table S2. Correlation between depressive-like behaviors and levels of lipid classes in the hippocampus**

| Lipids | Time in center  (OFT) | | Immobility time in FST | | Immobility time in TST | | Latency to feeding (NSFT) | |
| --- | --- | --- | --- | --- | --- | --- | --- | --- |
|  | *r* | *P* | *r* | *P* | *r* | *P* | *r* | *P* |
| PIP | -0.504 | 0.004 | 0.292 | 0.110 | 0.585 | 0.001 | 0.398 | 0.027 |
| PC | -0.016 | 0.933 | -0.194 | 0.295 | -0.021 | 0.909 | -0.273 | 0.137 |
| PI | -0.299 | 0.102 | 0.097 | 0.602 | 0.222 | 0.230 | 0.337 | 0.063 |
| PE | -0.342 | 0.059 | 0.104 | 0.577 | 0.535 | 0.002 | 0.142 | 0.446 |
| PS | -0.591 | <0.001 | 0.423 | 0.018 | 0.385 | 0.033 | 0.315 | 0.084 |
| PIP3 | 0.488 | 0.005 | -0.386 | 0.032 | -0.052 | 0.781 | -0.517 | 0.003 |
| PG | -0.239 | 0.196 | 0.046 | 0.804 | 0.031 | 0.869 | -0.076 | 0.686 |
| CL | -0.136 | 0.465 | -0.127 | 0.497 | -0.099 | 0.595 | -0.041 | 0.826 |
| LPC | -0.384 | 0.033 | 0.160 | 0.389 | 0.490 | 0.005 | 0.379 | 0.036 |
| LPE | -0.348 | 0.055 | 0.141 | 0.450 | 0.554 | 0.001 | 0.069 | 0.712 |
| LPI | -0.149 | 0.425 | -0.005 | 0.979 | -0.154 | 0.409 | 0.030 | 0.873 |
| PA | -0.207 | 0.264 | -0.083 | 0.657 | -0.063 | 0.736 | 0.214 | 0.249 |
| PIP2 | -0.169 | 0.364 | -0.139 | 0.454 | 0.116 | 0.534 | -0.210 | 0.257 |
| LPG | -0.263 | 0.153 | 0.006 | 0.976 | -0.180 | 0.333 | -0.202 | 0.275 |
| LPS | -0.397 | 0.027 | -0.026 | 0.888 | 0.057 | 0.762 | 0.141 | 0.450 |
| ZyE | 0.03 | 0.872 | -0.150 | 0.421 | -0.070 | 0.708 | -0.320 | 0.079 |
| ChE | -0.113 | 0.545 | -0.193 | 0.299 | -0.075 | 0.686 | -0.407 | 0.023 |
| StE | 0.274 | 0.136 | -0.244 | 0.186 | -0.257 | 0.163 | -0.239 | 0.195 |
| CerP | 0.24 | 0.194 | -0.253 | 0.170 | -0.453 | 0.010 | -0.482 | 0.006 |
| SM | 0.129 | 0.488 | -0.280 | 0.127 | -0.204 | 0.271 | -0.246 | 0.183 |
| Cer | -0.407 | 0.023 | 0.064 | 0.734 | 0.544 | 0.002 | 0.379 | 0.036 |
| ST | -0.048 | 0.797 | -0.032 | 0.863 | -0.067 | 0.719 | -0.066 | 0.725 |
| phSM | 0.335 | 0.065 | -0.412 | 0.021 | -0.455 | 0.010 | -0.451 | 0.011 |
| GM1 | -0.183 | 0.325 | -0.022 | 0.908 | -0.110 | 0.556 | 0.125 | 0.502 |
| GD2 | -0.154 | 0.407 | 0.051 | 0.787 | -0.122 | 0.513 | 0.103 | 0.582 |
| GM3 | -0.169 | 0.363 | -0.155 | 0.404 | -0.275 | 0.134 | 0.011 | 0.953 |
| GM2 | 0.389 | 0.031 | -0.368 | 0.042 | -0.648 | 0.000 | -0.431 | 0.016 |
| CerG2 | -0.081 | 0.665 | -0.117 | 0.531 | -0.304 | 0.096 | -0.029 | 0.878 |
| GD3 | 0.086 | 0.646 | -0.098 | 0.601 | -0.141 | 0.448 | -0.193 | 0.298 |
| CerG3 | -0.175 | 0.346 | -0.132 | 0.479 | 0.061 | 0.746 | 0.000 | 1.000 |
| OAHFA | -0.626 | <0.001 | 0.225 | 0.224 | 0.259 | 0.159 | 0.354 | 0.051 |
| AcCa | 0.255 | 0.166 | -0.438 | 0.014 | -0.239 | 0.195 | -0.348 | 0.055 |
| WE | -0.122 | 0.514 | -0.207 | 0.263 | -0.043 | 0.817 | -0.151 | 0.416 |
| DG | -0.336 | 0.064 | -0.077 | 0.679 | 0.068 | 0.718 | -0.008 | 0.964 |
| TG | -0.183 | 0.325 | 0.007 | 0.971 | -0.089 | 0.636 | -0.051 | 0.785 |
| MG | -0.152 | 0.416 | -0.285 | 0.120 | -0.242 | 0.190 | -0.419 | 0.019 |
| MGDG | -0.027 | 0.887 | -0.213 | 0.250 | -0.161 | 0.386 | -0.063 | 0.738 |
| SQDG | 0.273 | 0.137 | -0.422 | 0.018 | -0.346 | 0.057 | -0.203 | 0.273 |
| DGDG | -0.055 | 0.770 | -0.078 | 0.676 | -0.139 | 0.457 | -0.053 | 0.776 |
| SQMG | 0.256 | 0.165 | -0.617 | <0.001 | -0.297 | 0.105 | -0.602 | 0.001 |
| MGMG | 0.474 | 0.007 | -0.374 | 0.038 | -0.518 | 0.003 | -0.484 | 0.006 |
| Co | 0.052 | 0.780 | -0.059 | 0.754 | -0.009 | 0.961 | -0.076 | 0.683 |
