# Supplementary material for: Effects of (S)-ketamine on depression-like behaviors in a chronic variable stress model: a role of brain lipidome
Source: Front Cell Neurosci. 2023 Feb 15;17:1114914. doi: 10.3389/fncel.2023.1114914 (PMC9975603; doi:10.3389/fncel.2023.1114914)
Supplement: Supplementary file 3 [file Table_3.DOCX]

**Table S3. Correlation between depressive-like behaviors and levels of lipid classes in the prefrontal cortex**

| Lipids | Time in center  (OFT) | | Immobility time in FST | | Immobility time in TST | | Latency to feeding (NSFT) | |
| --- | --- | --- | --- | --- | --- | --- | --- | --- |
|  | *r* | *P* | *r* | *P* | *r* | *P* | *r* | *P* |
| PIP | -0.100 | 0.594 | 0.314 | 0.086 | 0.295 | 0.107 | 0.234 | 0.206 |
| PC | 0.063 | 0.737 | 0.069 | 0.713 | -0.067 | 0.719 | -0.161 | 0.386 |
| PE | -0.066 | 0.723 | 0.336 | 0.064 | 0.550 | 0.001 | 0.109 | 0.560 |
| PI | -0.353 | 0.051 | 0.380 | 0.035 | 0.495 | 0.005 | 0.265 | 0.149 |
| PG | 0.246 | 0.182 | 0.091 | 0.628 | 0.030 | 0.873 | -0.281 | 0.126 |
| PS | -0.099 | 0.594 | -0.017 | 0.926 | 0.329 | 0.071 | 0.055 | 0.770 |
| PIP3 | 0.310 | 0.089 | 0.077 | 0.680 | 0.029 | 0.877 | -0.178 | 0.338 |
| CL | 0.345 | 0.057 | 0.079 | 0.671 | 0.033 | 0.858 | -0.206 | 0.265 |
| LPC | -0.055 | 0.769 | 0.217 | 0.240 | 0.391 | 0.029 | 0.141 | 0.449 |
| LPE | 0.261 | 0.156 | 0.078 | 0.677 | 0.113 | 0.547 | -0.257 | 0.162 |
| LPG | 0.108 | 0.562 | 0.243 | 0.187 | 0.057 | 0.760 | -0.218 | 0.239 |
| LPI | 0.440 | 0.013 | -0.379 | 0.035 | -0.312 | 0.088 | -0.369 | 0.041 |
| PA | 0.221 | 0.232 | -0.251 | 0.173 | -0.054 | 0.772 | -0.372 | 0.039 |
| PIP2 | 0.380 | 0.035 | -0.294 | 0.108 | -0.190 | 0.305 | -0.351 | 0.053 |
| LPS | -0.018 | 0.922 | -0.087 | 0.643 | 0.339 | 0.062 | 0.005 | 0.978 |
| ZyE | 0.555 | 0.001 | -0.454 | 0.010 | -0.582 | 0.001 | -0.434 | 0.015 |
| ChE | 0.607 | 0.000 | -0.489 | 0.005 | -0.565 | 0.001 | -0.362 | 0.045 |
| StE | 0.402 | 0.025 | -0.378 | 0.036 | -0.569 | 0.001 | -0.482 | 0.006 |
| CerP | 0.240 | 0.194 | -0.253 | 0.170 | -0.453 | 0.010 | -0.482 | 0.006 |
| SM | 0.442 | 0.013 | -0.218 | 0.238 | -0.280 | 0.127 | -0.359 | 0.047 |
| Cer | 0.156 | 0.401 | -0.142 | 0.445 | 0.386 | 0.032 | 0.014 | 0.940 |
| ST | 0.142 | 0.447 | -0.172 | 0.356 | -0.042 | 0.822 | -0.201 | 0.278 |
| phSM | 0.426 | 0.017 | -0.232 | 0.210 | -0.363 | 0.045 | -0.348 | 0.055 |
| GM1 | 0.266 | 0.148 | -0.031 | 0.868 | -0.034 | 0.855 | -0.152 | 0.414 |
| GD2 | 0.323 | 0.076 | -0.025 | 0.894 | -0.134 | 0.473 | -0.179 | 0.335 |
| CerG2 | 0.308 | 0.092 | -0.299 | 0.102 | -0.220 | 0.235 | -0.118 | 0.528 |
| GM2 | -0.053 | 0.777 | 0.096 | 0.607 | -0.181 | 0.330 | -0.101 | 0.590 |
| GM3 | 0.491 | 0.005 | -0.215 | 0.245 | -0.315 | 0.085 | -0.292 | 0.111 |
| GD3 | 0.349 | 0.054 | -0.104 | 0.579 | -0.424 | 0.017 | -0.309 | 0.091 |
| CerG3 | -0.319 | 0.081 | -0.132 | 0.478 | 0.412 | 0.021 | -0.122 | 0.513 |
| OAHFA | -0.216 | 0.244 | -0.088 | 0.638 | 0.559 | 0.001 | 0.185 | 0.319 |
| WE | 0.329 | 0.070 | 0.084 | 0.653 | 0.201 | 0.277 | -0.278 | 0.129 |
| AcCa | 0.417 | 0.020 | -0.079 | 0.672 | -0.350 | 0.054 | -0.325 | 0.075 |
| DG | 0.311 | 0.089 | -0.091 | 0.625 | -0.104 | 0.577 | -0.324 | 0.076 |
| TG | 0.260 | 0.158 | 0.024 | 0.899 | 0.025 | 0.895 | -0.272 | 0.139 |
| MG | 0.219 | 0.236 | 0.023 | 0.900 | -0.086 | 0.644 | -0.350 | 0.054 |
| MGDG | 0.236 | 0.200 | 0.018 | 0.924 | -0.055 | 0.768 | -0.036 | 0.847 |
| SQDG | 0.308 | 0.092 | -0.132 | 0.479 | -0.181 | 0.329 | -0.329 | 0.071 |
| DGDG | 0.293 | 0.109 | -0.259 | 0.159 | -0.390 | 0.030 | -0.326 | 0.073 |
| SQMG | 0.306 | 0.094 | -0.264 | 0.151 | -0.519 | 0.003 | -0.264 | 0.151 |
| MGMG | 0.198 | 0.285 | -0.227 | 0.218 | -0.319 | 0.080 | -0.299 | 0.103 |
| Co | 0.204 | 0.270 | 0.056 | 0.766 | 0.279 | 0.129 | -0.134 | 0.471 |
