# Supplementary material for: Effects of (S)-ketamine on depression-like behaviors in a chronic variable stress model: a role of brain lipidome
Source: Front Cell Neurosci. 2023 Feb 15;17:1114914. doi: 10.3389/fncel.2023.1114914 (PMC9975603; doi:10.3389/fncel.2023.1114914)
Supplement: Supplementary file 4 [file Table_4.DOCX]

**Table S4. Characterization of lipids in the hippocampus**

|  | **LipidIon** | **Class** | Fold change | *P* value |
| --- | --- | --- | --- | --- |
| CVS+saline vs. Control | PC(36:4) | PC | 0.366 | 0.049 |
|  | WE(6:0/16:3) | WE | 0.502 | 0.023 |
|  | PC(18:1/24:0) | PC | 0.518 | 0.024 |
|  | PC(31:0/11:4) | PC | 0.529 | 0.033 |
|  | MGMG(16:0) | MGMG | 0.538 | 0.018 |
|  | SM(d36:2) | SM | 0.539 | 0.024 |
|  | PC(8:1e/11:3) | PC | 0.539 | 0.033 |
|  | PC(26:1/12:4) | PC | 0.541 | 0.013 |
|  | SM(d18:1/18:4) | SM | 0.548 | 0.039 |
|  | LPI(18:1) | LPI | 0.553 | 0.041 |
|  | WE(15:0/16:3) | WE | 0.554 | 0.049 |
|  | GD3(d38:2) | GD3 | 0.563 | 0.016 |
|  | MGDG(34:5) | MGDG | 0.568 | 0.010 |
|  | WE(8:0/18:3) | WE | 0.568 | 0.038 |
|  | SQDG(31:2e) | SQDG | 0.570 | 0.016 |
|  | DG(18:1/22:0) | DG | 0.572 | 0.024 |
|  | GM1(d36:2) | GM1 | 0.574 | 0.038 |
|  | phSM(t38:2) | phSM | 0.579 | 0.010 |
|  | SQMG(16:0) | SQMG | 0.579 | 0.024 |
|  | PC(18:0/20:2) | PC | 0.579 | 0.042 |
|  | WE(2:0/20:2) | WE | 0.587 | 0.035 |
|  | MGDG(40:2e) | MGDG | 0.592 | 0.033 |
|  | SM(d36:6) | SM | 0.593 | 0.030 |
|  | DGDG(43:10e) | DGDG | 0.595 | 0.023 |
|  | MGDG(18:1/24:0) | MGDG | 0.595 | 0.017 |
|  | SM(d34:2) | SM | 0.597 | 0.042 |
|  | SQDG(17:0/17:0) | SQDG | 0.598 | 0.010 |
|  | CerP(m19:0/15:0) | CerP | 0.603 | 0.010 |
|  | MGDG(18:1/24:1) | MGDG | 0.603 | 0.040 |
|  | AcCa(18:0) | AcCa | 0.604 | 0.013 |
|  | GM2(d34:5) | GM2 | 0.607 | 0.015 |
|  | MGDG(16:0/24:1) | MGDG | 0.611 | 0.016 |
|  | PC(16:2e/22:1) | PC | 0.612 | 0.040 |
|  | SM(d42:2) | SM | 0.614 | 0.030 |
|  | CL(21:0/22:6/16:1/18:1) | CL | 0.614 | 0.030 |
|  | GM1(d38:5) | GM1 | 0.615 | 0.024 |
|  | MGDG(38:3e) | MGDG | 0.620 | 0.024 |
|  | CL(87:5) | CL | 0.622 | 0.013 |
|  | CL(74:7) | CL | 0.624 | 0.023 |
|  | AcCa(18:1) | AcCa | 0.624 | 0.042 |
|  | StE(33:6) | StE | 0.625 | 0.038 |
|  | SM(d42:1) | SM | 0.626 | 0.027 |
|  | MGDG(16:0/26:0) | MGDG | 0.626 | 0.024 |
|  | MGDG(36:1e) | MGDG | 0.630 | 0.033 |
|  | MGDG(16:0/20:0) | MGDG | 0.630 | 0.013 |
|  | AcCa(16:0) | AcCa | 0.630 | 0.038 |
|  | MGDG(34:1e) | MGDG | 0.631 | 0.027 |
|  | Co(Q7) | Co | 0.631 | 0.010 |
|  | MGDG(16:0e/22:1) | MGDG | 0.632 | 0.023 |
|  | AcCa(14:0) | AcCa | 0.632 | 0.033 |
|  | CerG2GNAc1(d32:1) | CerG2GNAc1 | 0.633 | 0.016 |
|  | CL(83:5) | CL | 0.634 | 0.016 |
|  | CL(78:7) | CL | 0.634 | 0.016 |
|  | MGDG(16:0e/24:1) | MGDG | 0.634 | 0.024 |
|  | CerG2GNAc1(d34:4) | CerG2GNAc1 | 0.636 | 0.016 |
|  | MGDG(16:0/22:0) | MGDG | 0.637 | 0.024 |
|  | CL(23:0/16:0/22:1/22:6) | CL | 0.637 | 0.021 |
|  | SM(d18:2/21:3) | SM | 0.638 | 0.049 |
|  | PC(20:2/20:3) | PC | 0.639 | 0.023 |
|  | PG(20:0/22:6) | PG | 0.640 | 0.038 |
|  | SM(d18:1/24:2) | SM | 0.640 | 0.017 |
|  | DG(18:1/24:0) | DG | 0.641 | 0.024 |
|  | MGDG(32:0e) | MGDG | 0.642 | 0.024 |
|  | PG(42:0) | PG | 0.642 | 0.028 |
|  | PC(14:0/20:4) | PC | 0.642 | 0.033 |
|  | MGDG(44:4) | MGDG | 0.645 | 0.027 |
|  | MGDG(42:4) | MGDG | 0.646 | 0.033 |
|  | CL(17:0/20:0/18:0/22:4) | CL | 0.647 | 0.017 |
|  | MGDG(36:0e) | MGDG | 0.648 | 0.019 |
|  | SM(d41:1) | SM | 0.648 | 0.021 |
|  | PG(22:4/20:4) | PG | 0.649 | 0.021 |
|  | CL(76:8) | CL | 0.649 | 0.023 |
|  | MGDG(34:0e) | MGDG | 0.651 | 0.016 |
|  | PG(41:1) | PG | 0.653 | 0.042 |
|  | DG(18:1/24:1) | DG | 0.654 | 0.043 |
|  | DG(24:1/20:4) | DG | 0.654 | 0.041 |
|  | WE(6:0/16:2) | WE | 0.655 | 0.049 |
|  | DGDG(31:7) | DGDG | 0.656 | 0.016 |
|  | GM3(d36:1) | GM3 | 0.656 | 0.024 |
|  | CL(18:2/20:4/22:6/18:1) | CL | 0.657 | 0.024 |
|  | CL(80:12) | CL | 0.658 | 0.035 |
|  | CL(74:1) | CL | 0.658 | 0.042 |
|  | SM(d14:0/24:1) | SM | 0.663 | 0.022 |
|  | SM(d36:1) | SM | 0.664 | 0.024 |
|  | GM1(d36:1) | GM1 | 0.664 | 0.021 |
|  | WE(38:2) | WE | 0.665 | 0.042 |
|  | MGDG(40:0e) | MGDG | 0.665 | 0.028 |
|  | ST(d42:2) | ST | 0.665 | 0.024 |
|  | MGDG(38:2e) | MGDG | 0.666 | 0.026 |
|  | ST(d18:1/24:0) | ST | 0.666 | 0.023 |
|  | PA(48:4) | PA | 0.666 | 0.024 |
|  | SM(d34:0) | SM | 0.667 | 0.016 |
|  | LPC(14:0) | LPC | 1.528 | 0.038 |
|  | Cer(m18:1/18:2) | Cer | 1.636 | 0.024 |
|  | Cer(d17:1/18:0) | Cer | 1.666 | 0.024 |
|  | PE(16:0/20:4) | PE | 17.869 | 0.049 |
| CVS+es-Ket vs. CVS+saline | Cer(d17:1/18:0) | Cer | 0.618 | 0.003 |
|  | WE(6:0/16:2) | WE | 1.501 | 0.011 |
|  | SM(d34:1) | SM | 1.501 | 0.025 |
|  | PC(16:0/18:3) | PC | 1.502 | 0.019 |
|  | ChE(2:0) | ChE | 1.510 | 0.006 |
|  | SM(d42:2) | SM | 1.511 | 0.013 |
|  | DG(24:1/20:4) | DG | 1.515 | 0.014 |
|  | SM(d42:1) | SM | 1.531 | 0.041 |
|  | WE(44:4) | WE | 1.532 | 0.032 |
|  | SM(d36:6) | SM | 1.538 | 0.007 |
|  | AcCa(18:1) | AcCa | 1.542 | 0.014 |
|  | SM(d37:1) | SM | 1.560 | 0.013 |
|  | SM(d18:1/24:2) | SM | 1.566 | 0.048 |
|  | CL(76:4) | CL | 1.581 | 0.011 |
|  | MGMG(16:0) | MGDG | 1.605 | 0.027 |
|  | DG(18:1/24:0) | DG | 1.608 | 0.007 |
|  | AcCa(18:0) | AcCa | 1.612 | 0.011 |
|  | SQDG(31:2e) | SQDG | 1.626 | 0.030 |
|  | GM2(d34:5) | GM2 | 1.628 | 0.003 |
|  | WE(2:0/23:3) | WE | 1.630 | <0.001 |
|  | MGDG(18:1/24:1) | MGDG | 1.632 | 0.016 |
|  | WE(2:0/20:2) | WE | 1.634 | 0.025 |
|  | PC(20:1/18:1) | PC | 1.694 | 0.045 |
|  | PIP3(26:6) | PIP3 | 1.706 | 0.012 |
|  | WE(2:0/24:3) | WE | 1.760 | 0.019 |
|  | SQMG(16:0) | SQDG | 1.780 | 0.006 |
|  | PE(11:0/10:3) | PE | 1.796 | 0.011 |
|  | PE(10:0/11:4) | PE | 1.830 | 0.048 |
|  | SM(d18:1/18:4) | SM | 1.843 | 0.032 |
|  | PS(22:1e) | PS | 1.849 | 0.048 |
|  | SM(d18:2/21:3) | SM | 1.873 | 0.031 |
|  | WE(6:0/16:3) | WE | 1.876 | 0.006 |
|  | PE(17:0) | PE | 1.929 | 0.024 |
|  | AcCa(14:0) | AcCa | 1.937 | 0.011 |
|  | SM(d39:2) | SM | 2.026 | 0.030 |
|  | WE(8:0/18:3) | WE | 2.049 | 0.003 |
|  | WE(2:0/25:3) | WE | 2.054 | <0.001 |
|  | PE(9:0/10:1) | PE | 2.148 | 0.016 |
|  | PE(13:0/10:4) | PE | 2.373 | 0.015 |
|  | PE(9:0/12:4) | PE | 2.374 | 0.014 |
|  | AcCa(22:6) | AcCa | 2.469 | 0.007 |
|  | LPG(22:3) | LPG | 2.533 | 0.032 |
|  | PC(6:0/12:4) | PC | 2.570 | 0.040 |
|  | DG(19:3e) | DG | 2.593 | 0.012 |
|  | AcCa(16:1) | AcCa | 3.199 | 0.009 |
