# Supplementary material for: Effects of (S)-ketamine on depression-like behaviors in a chronic variable stress model: a role of brain lipidome
Source: Front Cell Neurosci. 2023 Feb 15;17:1114914. doi: 10.3389/fncel.2023.1114914 (PMC9975603; doi:10.3389/fncel.2023.1114914)
Supplement: Supplementary file 5 [file Table_5.DOCX]

**Table S5. Normalized lipids after ketamine treatment in the hippocampus and PFC**

|  | **LipidIon** | **Class** | **CVS+saline vs. Control** | | **CVS+es-Ket vs. CVS+saline** | |
| --- | --- | --- | --- | --- | --- | --- |
|  |  |  | **Fold change** | ***P* value** | **Fold change** | ***P* value** |
| Hippocampus | AcCa(14:0) | AcCa | 0.632 | 0.033 | 1.937 | 0.011 |
|  | AcCa(18:0) | AcCa | 0.604 | 0.013 | 1.612 | 0.011 |
|  | AcCa(18:1) | AcCa | 0.624 | 0.042 | 1.542 | 0.014 |
|  | DG(24:1/20:4) | DG | 0.654 | 0.041 | 1.515 | 0.014 |
|  | GM2(d34:5) | GM2 | 0.607 | 0.015 | 1.628 | 0.003 |
|  | MGDG(18:1/24:1) | MGDG | 0.603 | 0.040 | 1.632 | 0.016 |
|  | MGMG(16:0) | MGMG | 0.538 | 0.018 | 1.605 | 0.027 |
|  | SM(d18:1/18:4) | SM | 0.548 | 0.039 | 1.843 | 0.032 |
|  | SM(d18:1/24:2) | SM | 0.640 | 0.017 | 1.566 | 0.048 |
|  | SM(d18:2/21:3) | SM | 0.638 | 0.049 | 1.873 | 0.031 |
|  | SM(d36:6) | SM | 0.593 | 0.030 | 1.538 | 0.007 |
|  | SM(d42:1) | SM | 0.626 | 0.027 | 1.531 | 0.041 |
|  | SQDG(31:2e) | SQDG | 0.570 | 0.016 | 1.626 | 0.030 |
|  | SQMG(16:0) | SQMG | 0.579 | 0.024 | 1.780 | 0.006 |
|  | WE(2:0/20:2) | WE | 0.587 | 0.035 | 1.634 | 0.025 |
|  | WE(6:0/16:2) | WE | 0.655 | 0.049 | 1.501 | 0.011 |
|  | WE(6:0/16:3) | WE | 0.502 | 0.023 | 1.876 | 0.006 |
|  | WE(8:0/18:3) | WE | 0.568 | 0.038 | 2.049 | 0.003 |
| PFC | ChE(2:0) | ChE | 0.636 | 0.012 | 1.897 | 0.006 |
|  | ChE(30:0) | ChE | 0.597 | 0.003 | 1.636 | 0.019 |
|  | SM(d36:2) | SM | 0.609 | 0.001 | 1.504 | 0.019 |
|  | ZyE(20:5) | ZyE | 0.614 | 0.004 | 1.645 | 0.019 |
|  | ZyE(35:6) | ZyE | 0.386 | <0.001 | 2.029 | 0.003 |
