# Supplementary material for: Effects of (S)-ketamine on depression-like behaviors in a chronic variable stress model: a role of brain lipidome
Source: Front Cell Neurosci. 2023 Feb 15;17:1114914. doi: 10.3389/fncel.2023.1114914 (PMC9975603; doi:10.3389/fncel.2023.1114914)
Supplement: Supplementary file 6 [file Table_6.DOCX]

**Table S6. Correlation between depressive-like behaviors and levels of lipid species in the hippocampus**

| **LipidIon** | **Time in center**  **(OFT)** | | **Immobility time in FST** | | **Immobility time in TST** | | **Latency to feeding (NSFT)** | |
| --- | --- | --- | --- | --- | --- | --- | --- | --- |
|  | ***r*** | ***P*** | ***r*** | ***P*** | ***r*** | ***P*** | ***r*** | ***P*** |
| AcCa(14:0) | 0.496 | 0.026 | -0.322 | 0.166 | -0.574 | 0.008 | -0.225 | 0.339 |
| AcCa(18:0) | 0.496 | 0.026 | -0.267 | 0.256 | -0.542 | 0.013 | -0.218 | 0.357 |
| AcCa(18:1) | 0.509 | 0.022 | -0.310 | 0.183 | -0.553 | 0.011 | -0.201 | 0.396 |
| DG(24:1/20:4) | 0.687 | 0.001 | -0.309 | 0.185 | -0.652 | 0.002 | -0.336 | 0.148 |
| GM2(d34:5) | 0.580 | 0.007 | -0.257 | 0.275 | -0.707 | 0.000 | -0.288 | 0.218 |
| MGDG(18:1/24:1) | 0.610 | 0.004 | -0.065 | 0.784 | -0.629 | 0.003 | -0.175 | 0.462 |
| MGMG(16:0) | 0.513 | 0.021 | -0.232 | 0.324 | -0.691 | 0.001 | -0.418 | 0.066 |
| SM(d18:1/18:4) | 0.717 | 0.000 | -0.156 | 0.511 | -0.714 | <0.001 | -0.312 | 0.180 |
| SM(d18:1/24:2) | 0.452 | 0.046 | -0.156 | 0.512 | -0.703 | 0.001 | -0.114 | 0.633 |
| SM(d18:2/21:3) | 0.452 | 0.045 | -0.127 | 0.593 | -0.589 | 0.006 | -0.239 | 0.310 |
| SM(d36:6) | 0.268 | 0.254 | -0.144 | 0.545 | -0.470 | 0.036 | 0.008 | 0.974 |
| SM(d42:1) | 0.489 | 0.029 | -0.148 | 0.534 | -0.640 | 0.002 | -0.123 | 0.604 |
| SQDG(31:2e) | 0.404 | 0.077 | -0.534 | 0.015 | -0.683 | 0.001 | -0.197 | 0.406 |
| SQMG(16:0) | 0.532 | 0.016 | -0.392 | 0.087 | -0.523 | 0.018 | -0.429 | 0.059 |
| WE(2:0/20:2) | 0.339 | 0.143 | -0.414 | 0.070 | -0.505 | 0.023 | -0.001 | 0.996 |
| WE(6:0/16:2) | 0.639 | 0.002 | -0.199 | 0.401 | -0.625 | 0.003 | -0.142 | 0.551 |
| WE(6:0/16:3) | 0.508 | 0.022 | -0.447 | 0.048 | -0.478 | 0.033 | -0.339 | 0.144 |
| WE(8:0/18:3) | 0.534 | 0.015 | -0.166 | 0.485 | -0.588 | 0.006 | -0.205 | 0.386 |
