# Supplementary material for: Effects of (S)-ketamine on depression-like behaviors in a chronic variable stress model: a role of brain lipidome
Source: Front Cell Neurosci. 2023 Feb 15;17:1114914. doi: 10.3389/fncel.2023.1114914 (PMC9975603; doi:10.3389/fncel.2023.1114914)
Supplement: Supplementary file 7 [file Table_7.DOCX]

**Table S7. Characterization of lipids in the PFC**

|  | **LipidIon** | **Class** | **Fold change** | ***P* value** |
| --- | --- | --- | --- | --- |
| CVS+saline vs. Control | PC(6:0/12:4) | PC | 0.359 | 0.037 |
|  | ZyE(35:6) | ZyE | 0.386 | <0.001 |
|  | PC(36:4) | PC | 0.400 | 0.022 |
|  | SM(d44:2) | SM | 0.410 | 0.005 |
|  | SM(d32:1) | SM | 0.469 | 0.003 |
|  | SM(d18:0/22:1) | SM | 0.510 | 0.027 |
|  | SM(d18:1/24:2) | SM | 0.514 | 0.005 |
|  | SM(d18:1/18:4) | SM | 0.520 | 0.003 |
|  | MG(32:0) | MG | 0.527 | 0.014 |
|  | SM(d18:1/23:0) | SM | 0.527 | 0.004 |
|  | PC(18:1/18:1) | PC | 0.527 | 0.004 |
|  | SM(d33:1) | SM | 0.531 | 0.005 |
|  | SM(d34:2) | SM | 0.541 | 0.003 |
|  | ChE(22:6) | ChE | 0.571 | 0.003 |
|  | StE(33:6) | StE | 0.576 | 0.005 |
|  | PC(25:0/11:1) | PC | 0.582 | 0.039 |
|  | PC(20:1/18:1) | PC | 0.585 | 0.041 |
|  | SM(t34:1) | SM | 0.591 | 0.023 |
|  | ZyE(21:5) | ZyE | 0.593 | 0.004 |
|  | MGMG(16:0) | MGMG | 0.597 | 0.029 |
|  | ChE(30:0) | ChE | 0.597 | 0.003 |
|  | PC(34:1e) | PC | 0.602 | 0.037 |
|  | PC(34:0e) | PC | 0.602 | 0.042 |
|  | SM(d38:0) | SM | 0.605 | 0.030 |
|  | SM(d36:2) | SM | 0.609 | 0.001 |
|  | ZyE(20:5) | ZyE | 0.614 | 0.004 |
|  | SM(d41:1) | SM | 0.627 | 0.031 |
|  | SQMG(16:0) | SQMG | 0.630 | 0.025 |
|  | ChE(2:0) | ChE | 0.636 | 0.012 |
|  | SM(d34:0) | SM | 0.636 | 0.003 |
|  | MG(34:0) | MG | 0.639 | 0.037 |
|  | SM(d35:1) | SM | 0.645 | 0.005 |
|  | GM1(d38:5) | GM1 | 0.646 | 0.024 |
|  | PG(41:1) | PG | 0.648 | 0.022 |
|  | SM(d18:1/23:3) | SM | 0.651 | 0.028 |
|  | ST(t18:0/18:0) | ST | 0.655 | 0.049 |
|  | MGDG(16:0/22:0) | MGDG | 0.660 | 0.044 |
|  | SM(d37:1) | SM | 0.666 | 0.037 |
|  | PI(16:0/20:5) | PI | 1.504 | 0.024 |
|  | PI(18:1/22:6) | PI | 1.504 | 0.028 |
|  | PI(10:0/20:4) | PI | 1.505 | 0.012 |
|  | PI(20:1/20:4) | PI | 1.507 | 0.015 |
|  | PI(16:0/22:6) | PI | 1.529 | 0.023 |
|  | PI(20:4/20:4) | PI | 1.535 | 0.012 |
|  | PI(16:0/20:4) | PI | 1.546 | 0.020 |
|  | PI(16:0/16:0) | PI | 1.550 | 0.038 |
|  | PI(18:1e/20:4) | PI | 1.553 | 0.011 |
|  | PI(16:0e/20:4) | PI | 1.579 | 0.022 |
|  | PE(9:0/10:1) | PE | 1.610 | 0.047 |
|  | PI(16:0/20:3) | PI | 1.625 | 0.014 |
|  | PE(20:1p/22:4) | PE | 1.650 | 0.042 |
|  | PI(16:0/22:4) | PI | 1.672 | 0.015 |
|  | PI(18:2/20:4) | PI | 1.701 | 0.008 |
|  | PI(16:0/18:1) | PI | 1.707 | 0.007 |
|  | PE(13:0/10:4) | PE | 1.780 | 0.015 |
|  | PE(9:0/12:4) | PE | 1.783 | 0.016 |
|  | LPC(16:2e) | LPC | 1.794 | 0.007 |
|  | PE(11:0/10:1) | PE | 1.852 | 0.049 |
|  | PE(18:1e/20:4) | PE | 1.852 | 0.015 |
|  | LPC(24:0) | LPC | 2.105 | 0.005 |
|  | Cer(m44:3) | Cer | 2.197 | 0.005 |
| CVS+es-Ket vs. CVS+saline) | PI(18:0/20:3) | PI | 0.291 | 0.019 |
|  | PE(18:2e/20:1) | PE | 0.640 | 0.019 |
|  | ZyE(35:6) | ZyE | 2.029 | 0.003 |
|  | ChE(2:0) | ChE | 1.897 | 0.006 |
|  | SM(d36:2) | SM | 1.504 | 0.019 |
|  | AcCa(20:4) | AcCa | 1.512 | 0.019 |
|  | ChE(30:0) | ChE | 1.636 | 0.019 |
|  | ZyE(20:5) | ZyE | 1.645 | 0.019 |
