# Supplementary material for: Effects of (S)-ketamine on depression-like behaviors in a chronic variable stress model: a role of brain lipidome
Source: Front Cell Neurosci. 2023 Feb 15;17:1114914. doi: 10.3389/fncel.2023.1114914 (PMC9975603; doi:10.3389/fncel.2023.1114914)
Supplement: Supplementary file 8 [file Table_8.DOCX]

**Table S8. Correlation between depressive-like behaviors and levels of lipid species in the PFC**

| **LipidIon** | **Time in center**  **(OFT)** | | **Immobility times (TST)** | | **Immobility time (FST)** | | **Latency to feeding (NSFT)** | |
| --- | --- | --- | --- | --- | --- | --- | --- | --- |
|  | ***r*** | ***P*** | ***r*** | ***P*** | ***r*** | ***P*** | ***r*** | ***P*** |
| ChE(2:0) | 0.540 | 0.014 | -0.480 | 0.032 | -0.703 | 0.001 | -0.203 | 0.390 |
| ChE(30:0) | 0.646 | 0.002 | -0.721 | <0.001 | -0.756 | <0.001 | -0.261 | 0.267 |
| SM(d36:2) | 0.570 | 0.009 | -0.413 | 0.070 | -0.656 | 0.002 | -0.266 | 0.258 |
| ZyE(20:5) | 0.585 | 0.007 | -0.683 | 0.001 | -0.701 | 0.001 | -0.294 | 0.208 |
| ZyE(35:6) | 0.675 | 0.001 | -0.650 | 0.002 | -0.761 | <0.001 | -0.385 | 0.094 |
