# Supplementary material for: Effects of (S)-ketamine on depression-like behaviors in a chronic variable stress model: a role of brain lipidome
Source: Front Cell Neurosci. 2023 Feb 15;17:1114914. doi: 10.3389/fncel.2023.1114914 (PMC9975603; doi:10.3389/fncel.2023.1114914)
Supplement: Supplementary file 9 [file Table_9.DOCX]

**Table S9. Effect of CVS on the fatty acid composition in the hippocampus and prefrontal cortex**

| Number of carbons | Hippocampus | | prefrontal cortex | |
| --- | --- | --- | --- | --- |
|  | *F _(2,28)_* | *P* | *F _(2,28)_* | *P* |
| <16 | / | / | 4.056 | 0.028 |
| <17 | 16.06 | <0.001 | / | / |
| 19 | 3.764 | 0.036 | 1.003 | 0.380 |
| 21 | 6.873 | 0.004 | 6.931 | 0.004 |
| 23 | 11.04 | <0.001 | 4.087 | 0.028 |
| 30 | 2.316 | 0.117 | 16.63 | <0.001 |
| 31 | 4.257 | 0.024 | 3.136 | 0.059 |
| 32 | 3.593 | 0.041 | 1.144 | 0.333 |
| 35 | 0.877 | 0.427 | 9.745 | <0.001 |
| 37 | 1.129 | 0.338 | 3.441 | 0.046 |
| 40 | 3.495 | 0.042 | 0.753 | 0.480 |
| 41 | 4.161 | 0.026 | 2.563 | 0.095 |
| >41 | 2.940 | 0.069 | 1.334 | 0.280 |
| Number of double bonds | *F _(2,28)_* | *P* | *F _(2,28)_* | *P* |
| 0 | 12.93 | <0.001 | 1.993 | 0.155 |
| 1 | 1.319 | 0.283 | 1.565 | 0.227 |
| ≥2 | 3.077 | 0.062 | 0.334 | 0.717 |
